# Post Bellum Problems

**Published:** 1918-08

**Authors:** 


### Post Bellum Problems.

If, in time of peace, a country should prepare for war, it is equally important, in time of war, to prepare for peace. Such preparation by no means implies an excess of optimism as to the date when the war will end, though it may be said that it is scarcely conceivable that it will last too long to allow delay in beginning the discussion of the major problems of readjustment to peace conditions.

SHALL STANDARDS OF MEDICAL EDUCATION BE REDUCED? This is not an academic question but one which has been seriously raised. According to standards of graduation long in vogue among our Allies, the war almost immediately reduced the supply of physicians to a minimum for the civil population and has already left even the armies with a relative shortage, though this has been compensated by various means, including a supply from our own formerly redundant profession. Even in our own country, the supply of military medical men by volunteering has just about reached the limit of purely voluntary action, concomitant with the raising of the force at first announced (2 million) and the additional contingent already provided for.

The temptation is strong, therefore, to stimulate the production of more physicians by lowering educational standards and shortening the period of medical study, even if the actual amount of work is not diminished. From the purely military standpoint, even a genuine lowering of standards of technical instruction would be justified, as a large part of the work of army surgeons could be arranged so as to assign to any individual the repetition of a comparatively simple task with ample provision not only for perfecting him in its performance but with the necessary discipline to prevent his essaying other work beyond his ability. Moreover, the military service includes both clinical and didactic instruction which would bring even an imperfectly educated physician up to the requisite standard for civil practice, in a period of a year or two.

However, it is a safe rule always to suspect unworthy motives behind any plea for degrading medical standards and military necessity should not be conceded as a valid argument until it is presented by proper authority, in unmistakable terms. Here is one point in regard to which there should be no anticipation of necessity.

With any such duration of the war as now seems at all probable, there will be comparatively little actual wastage of physicians—probably not much over 2% per annum, and indeed, not much if any more than the mortality of the older men remaining in civil practice. Reconstruction work on returned soldiers will probably be just about balanced by the greater health of the remainder of the army and their acquired training in hygiene, immunization against typhoid, etc. With the almost complete cessation of immigration —representing fully half of the previous increase of our population—for a period of at least five years, the slight but still appreciable check on births, the actual loss by death in the army, and, more important than all else, the adjustment of civil practice in its various manifestations including hospital service, to a much smaller staff, the sudden return of 1 physician to about 100 men, will obviously disturb the economic relations of profession and laity. On the other hand, there will have been, according to the tendency of increasing educational requirements for the last decade or so, a gradual reduction of physicians to total population and there will probably be a greater, relatively permanent withdrawal of physicians from private to public duties than of men from civil to military professional care. Just how ratios will balance against the presumptive diminution of sickness and the increased efficiency of professional distribution, can be determined only by experience. But, unless the war is protracted far beyond the time now generally regarded as possible, it is plain that very serious economic detriment would result to the profesion from the attempt to increase medical graduation by lowering standards. In short, most of the good that has been accomplished by ten years of hard work and skillful management would be lost. It should also be considered that no hastening of graduation and increase of students that would be even tolerable from the ultimate standpoint of securing men that would deserve the title of physician, could operate immediately to relieve the necessity of still further drawing on the existing personnel of the profession to any marked degree. Such a process could not yield any appreciable increase of available surgeons for almost a year, nor reach the maximum indicated by military needs within four years and it is doubtful if it could be checked within four years more. A very slight additional pressure to secure volunteers from the extant profession would secure better results for at least two years and it is scarcely to be doubted that the war will either be over in that time or a better estimate of its duration and needs can be made than at present.

---

**New Mercurial.** Louis Bory and Albert Jacquet, Le Prog. Med., Dec. 1, 1917, report favorably on ortho-amido-benzoate of mercury, dose 4-6 c.g. a day.

